# Macrophage-Mediated Immune Responses: From Fatty Acids to Oxylipins

**DOI:** 10.3390/molecules27010152

**Published:** 2021-12-28

**Authors:** Barbara Balestrieri, David Di Costanzo, Daniel F. Dwyer

**Affiliations:** Department of Medicine, Division of Allergy and Clinical Immunology, Brigham and Women’s Hospital, Harvard Medical School, Boston, MA 02115, USA; ddicostanzo@bwh.harvard.edu (D.D.C.); dfdwyer@bwh.harvard.edu (D.F.D.)

**Keywords:** fatty acids, oxylipins, macrophages

## Abstract

Macrophages have diverse functions in the pathogenesis, resolution, and repair of inflammatory processes. Elegant studies have elucidated the metabolomic and transcriptomic profiles of activated macrophages. However, the versatility of macrophage responses in inflammation is likely due, at least in part, to their ability to rearrange their repertoire of bioactive lipids, including fatty acids and oxylipins. This review will describe the fatty acids and oxylipins generated by macrophages and their role in type 1 and type 2 immune responses. We will highlight lipidomic studies that have shaped the current understanding of the role of lipids in macrophage polarization.

## 1. Introduction

Macrophages exist in virtually every tissue. Ontogenetically, macrophages deriving from the primitive yolk sac, home to various tissues, including the brain and fetal liver [1]. The second wave of proliferation originates from fetal liver seeding remaining tissues, including bone marrow [2]. This process defines the first line of heterogeneity. An additional diversification of macrophages happens in the particular niche in which they develop and it is due to the stimulation by environmental cues and pathogens. Thus, tissue macrophages acquire peculiar characteristics, including lipid composition [3,4].

Another level of complexity in the study of macrophages is due to the activation in vitro of bone-marrow-derived (BM)-macrophages, or human monocyte-derived macrophages, which resemble in vivo counterparts, mirroring T helper (Th)1 (type 1) and Th2 cell (type 2) activation. Classically activated macrophages or M1 are generated by exposure to type 1 stimuli, including LPS and IFNγ, and produce IL-12, IL-1β, TNF-α, and the chemokines CXCL10, CXCL9, and CXCL11. M2 macrophages are activated by type 2 cytokines, particularly IL-4, the prototypical stimulus for alternative activated macrophages. M2 macrophages express CD206, arginase-1 (Arg-1), and produce IL-13, IL-33, and the chemokines CCL22 and CCL21 [5]. Although it is helpful to classify macrophages into different groups, it is likely that in vivo differentially activated macrophages coexist, continuously changing depending on the microenvironment in which they develop.

Macrophages are a rich source of bioactive lipids, including fatty acids (FAs) and their oxygenated metabolites oxylipins. FAs are characterized by a carbon backbone of various lengths. They are classified based on their degree of saturation in saturated fatty acids (SFAs, no double bonds), mono- or polyunsaturated (MUFAs or PUFAs, one or more double bonds, respectively). FAs may act directly at cognate receptors in an autocrine or paracrine fashion. They can bind specific G-protein-coupled receptors (GPCRs), including free fatty acid receptors (FFARs)1-4 and GPR84, expressed on macrophages and target cells [6,7,8], thus serving as signaling molecules [9,10].

**Figure 1:**
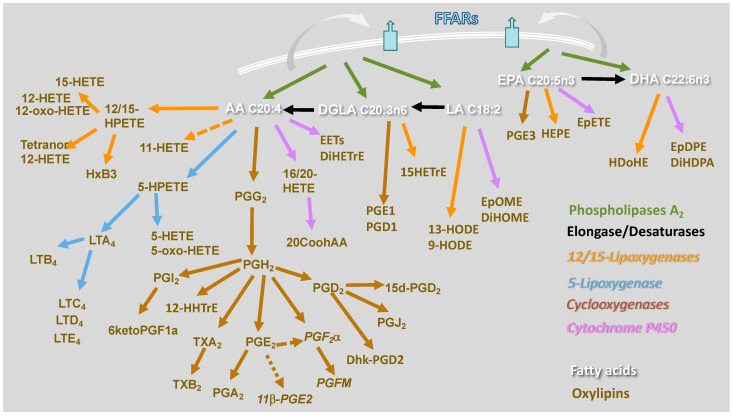
Enzymatic pathways and oxylipins derived from the omega-6 FAs, arachidonic acid (AA), di-homo-gamma-linolenic acid (DGLA), and linoleic acid (LA), and the omega-3 FAs, eicosapentaenoic acid (EPA) and docosahexaenoic acid (DHA). Enzymatic pathways are color coded. Phospholipases A_2_ are depicted in green, elongases/desaturases in black, 12/15-lipoxygenase in orange, 5-lipoxygenase in blue, cyclooxygenases in brown, cytochrome P450 in purple. 20-carboxyAA (20CooHAA), dihydroxy-eicosatrienoic acid (DiHETrE), dihydroxy-docosa-pentaenoic acid (DiHDPA), 13,14-dihydro-15-keto prostaglandin D_2_ (Dhk-PGD_2_), dihydroxy-octadecenoic acid (DiHOME), epoxy-docosapentaenoic acid (EpDPE), epoxy-eicosatrienoic acid (EET), epoxy-octadecenoic acid (EpOME), hydroxy-eicosatrienoic acid (HETrE), hydroxy-docosahexaenoic acid (HDoHE), hydroxy-eicosatetraenoic acid (HETE), 5-oxo-eicosatetraenoic acid (5-oxo-HETE), hydroperoxy-eicosatetraenoic acid (HPETE), hydroxy-eicosapentaenoic acid (HEPE), hepoxilin B3 (HXB3), 12-hydroxy-heptadecatrenoic acid (12-HHTrE), hydroxy-octadecadienoic acid (HODE), leukotriene A_4_ (LTA_4_), leukotriene B_4_ (LTB_4_), leukotriene C_4_ (LTC_4_), leukotriene D_4_ (LTD_4_), leukotriene E_4_ (LTE_4_), prostaglandin H_2_ (PGH_2_), prostaglandin E_2_ (PGE_2_), prostaglandin D_2_ (PGD_2_), prostaglandin F metabolite (PGFM), and thromboxane B_2_ (TxB_2_).

Macrophages incorporate and utilize FAs through several pathways. Linoleic acid (LA, C18:0 N6) and alpha-linolenic acid (ALA, C18:3 N3) are essential fatty acids that mammalian cells cannot synthesize. Therefore, they are provided to macrophages either through lipoproteins or as non-esterified fatty acids bound to albumin. Then, they are transported intracellularly via CD36 or fatty acid transporters (FATs), respectively, and shuttled intracellularly to various organelles via fatty acid-binding protein (FABP)-4 and 5 [11,12]. Phagocytosis and efferocytosis also contribute to enriching the FA repertoire of macrophages. MUFAs and PUFAs are synthesized in the cell from LA and ALA by the action of fatty acid elongase (ELOVL) and desaturase (FADS) enzymes [13]. Through acetyl-CoA synthases (ACSs), which also serve as FA transporters [14], FAs may enter the cell and be esterified into ceramide, phospholipids, and triglyceride, therefore performing many functions [15]. FAs incorporated into neutral lipids such as triglycerides are a source of stored energy, or FAs can be incorporated into structural lipids such as membrane phospholipids, sphingolipids, and plasmalogens. Additionally, FAs present in the second position of membrane phospholipids are hydrolyzed by phospholipase A_2_ (PLA_2_) enzymes [16,17]. PUFAs liberated from membrane phospholipids by PLA_2_ are metabolized into oxylipins through three major enzymatic pathways, cyclooxygenase (COX), lipoxygenase (LOX), and cytochrome P450 (CYP450), and other nonenzymatic pathways. Each pathway comprises multiple enzymes that produce several bioactive lipid mediators (Figure 1).

The first comprehensive mass spectrometry lipid analysis of macrophages was conducted on RAW264.7 cells using an M1-like stimulation with Kdo2-lipid A (KLA), a toll-like receptor 4 (TLR4) agonist [18]. The report correlated the changes in lipids induced by KLA to gene expression. Remarkably, the generation of FAs and oxylipins correlated with the changes in the expression of PLA_2_s and other enzymes involved in the production of oxylipins. KLA induced the increased expression of several enzymes, including group V PLA_2_ (Pla2g5), prostaglandin-endoperoxide synthase 2 (Ptgs2), also known as cyclooxygenase-2 (COX-2), and microsomal prostaglandin E synthase 2 (Pges2), which correlated with the increase in prostaglandin (PG)E_2_, PGF_2_, PGD_2_, and its metabolites PGJ_2_, 15-deoxy-12,14-PGD_2_, and 15-deoxy-12,14-PGJ_2_. Interestingly, unsaturated fatty acids were reduced, especially at 24 h stimulation, likely due to conversion to oxylipins. Indeed, KLA, similarly to the effects of other TLRs [19], increased PGD_2_ > PGE_2_ > PGF_2_α, but not cysteinyl leukotrienes (CysLTs). However, a shorter stimulation of less than 60 min was sufficient to induce Ca^++^ flux and leukotriene (LT)C_4_ release [19]. These effects were likely dependent on subcellular lipid distribution [20] or specific transcriptomic and metabolomic programs induced in M1-polarized macrophages, for instance, the regulation of the TCA pathway, which is suppressed in M1 and activated in M2 macrophages [21].

Thus, FAs serve multiple purposes in the cell: they contribute to the membrane structure; they are a source of energy; and they are second messengers and signaling molecules in immune responses, directly or indirectly through the generation of oxylipins. This review will summarize FAs and derived oxylipins produced by macrophages as revealed by comprehensive lipidomic studies and we will correlate them with the functions of M1 and M2 macrophages in type 1 and type 2 immune responses.

## 2. Fatty Acids

FAs have different activities based on the degree of saturation (SFAs, MUFAs, and PUFAs) and the length of the fatty acid chain, medium-chain FAs having 12–15 carbons and long-chain FAs containing 16 to 26 carbons. Unsaturated FAs are further classified based on the position of the final double bond at n-9, n-6, or n-3 from the methyl end, which define, respectively, omega-9, omega-6, or omega-3 FAs (Table 1).

### 2.1. Medium-Chain and Uneven Saturated Fatty Acids

Medium-chain (MC)SFAs include lauric acid (LU, C12:0), myristic acid (MA, C14:0), and pentadecanoic (PdA, C15:0). One of the least abundant FAs in macrophages is MA, which has a prominent function in post-translational modification of proteins carrying the amino acids methionine-glycine (MG) at the N-terminus. The N-myristoleic enzymes 1 and 2 are expressed in macrophages, where targeted proteins are directed to lipid reach subcellular structures such as Golgi and caveolae. Myristoylation increases type 1 immune responses [22,23] (Table 2). Lauric acid (LU, C12:0) composes 45% coconut oil. LU has proinflammatory functions in type 1 inflammation [24,25,26,27,28] (Table 2). However, the function of MA and LU in M2 macrophages and type 2 immune responses are less known.

Pentadecanoic acid (PdA, C15:0) and heptadecanoic acid or margaric acid (MaA, C17:0) are uneven SFAs found in dairy products [29]. Recent reports suggest a protective effect of PdA in type 1 inflammation [29,30] (Table 2).

### 2.2. Long-Chain Saturated Fatty Acids

Long-chain (LC)SFAs are essential in macrophage development, polarization and, type 1 inflammation [21,31]. Particularly, PA (C16:0) and stearic acid (SA, C18:0) increase during macrophage differentiation [32]. Furthermore, LCSFAs can induce macrophage activation and cytokine release in M1 conditions. Indeed, levels of LCSFAs are increased in the serum of patients with type 2 diabetes, cardiovascular diseases, and obesity, pathologies in which macrophages have a prominent pathogenetic role. These effects have been linked to palmitate-induced endoplasmic reticulum (ER) stress, which, in macrophages, is dependent on the presence of fatty acid-binding protein 4 (FABP4) and can be prevented by unsaturated FAs [32,33]. An increase in LCSFAs has also been reported in HIV patients, likely contributing to HIV pathogenesis due to SA and PA activation of human monocytes [34].

PA is introduced into the cells from the diet. Once in the cell, PA is metabolized into lysophosphatidylcholine, diacylglycerol, and ceramide. PA exerts its proinflammatory functions in macrophages by activating TLR2 and TLR4 through direct and indirect mechanisms. In THP-1 monocyte-macrophages, PA increased the secretion of IL-1β, TNF-α, and IL-8 by activating the nucleotide-binding oligomerization domain, leucine-rich repeat and pyrin domain-containing 3 (NLRP3) inflammasome, and through ceramide production. At the same time, LA and OA reduced IL-1β secretion not by reducing its synthesis but by reducing its processing by the NLRP3 inflammasome [35,36]. In BM-macrophages, PA upregulated the channel forming molecule pannexin-1 allowing the release of nucleotides attracting neutrophils [37] and increased LPS-induced production of IL-6, TNF-α, and IL1-β [38]. PA binds the FFAR1 or GPR40 [39,40]. Indeed, in HEK293, PA acted as a partial β-arrestin agonist and a Gq agonist [41]. However, FFAR-1 is the receptor for other medium- to long-chain FAs expressed in macrophages, neutrophils, and muscular cells [39], and it is also highly expressed in beta cells, where it regulates insulin secretion [40]. Therefore, the functions of PA in vivo may reflect the effects of PA on multiple cells.

Macrophage development involves the rearrangement of the lipids composing the plasma membrane [21,31,42]. SA has been reported to accumulate in the supernatant of human monocyte-derived macrophages [43]. Its effects included increased maturation of macrophages and shifting macrophages toward a classical M1 phenotype. Furthermore, mouse BM-macrophages cultured with SA acquired a more mature phenotype based on increased CD11c expression, a feature not shared with LA and OA [35]. Mouse peritoneal macrophages activated by SA increase the production of proinflammatory cytokines, including IL-1α, TNF-α, and ER stress, leading to apoptosis [44]. Furthermore, like PA, SA activated NLRP3 by creating intracellular crystals [45].

Arachidic acid (ArA, C20:0), and other very-long-chain SFAs (VLCSFAs) docosanoic acid (C22:0), tricosanoic acid (C23:0), tetracosanoic acid (C24:0), and hexacosanoic acid (C26:0) are derived from elongation of SA or PA or from the diet. Several studies have suggested a potential inverse correlation between these circulating VLCSFAs and the risk of cardiovascular disease [46,47,48]. However, ArA may target TLR4 to induce a proinflammatory response and ER stress, particularly in macrophages, contributing to obesity [49]. Therefore, further studies are needed to understand the mechanisms underlying these associations and the specific role of each VLCSFA in macrophage activation and type 2 inflammation.

### 2.3. Monounsaturated Fatty Acids

Palmitoleic acid (PoA, C16:1) is synthesized from PA and modulates type 1 inflammation. Indeed, PoA counteracted the effect of PA on the J774A.1 mouse macrophage cell line by reducing the secretion of TNF-α and the expression of COX-2 and TLR2. Furthermore, resident peritoneal macrophages from Swiss male mice released PoA following stimulation with zymosan, suggesting that PoA or its metabolites [50] could exert an anti-inflammatory function in the contest of type 1 inflammation [51]. Others reported inhibitory functions of PoA, including prevention of PA-induced NF-kB activation; reduced expression of type 1 inflammatory molecules, including IL-6, IL-12β, and Nos2 in BM-macrophages fed a high-fat diet [52]; and the increased expression of molecules associated with type 2 inflammation, including Mannose receptor C-type 1 (Mrc1), IL-10, and Transforming growth factor beta-1 (Tgf-β1). Furthermore, administration of PoA in vivo increased M2 polarization of liver macrophages and reduced insulin resistance in mice fed a high-fat diet [53].

Oleic acid (OA, C18:1) is a dietary lipid that may exert opposing proinflammatory or anti-inflammatory functions depending on whether additional stimuli or environmental factors are involved. In vitro, OA reduced inflammasome activation in mouse peritoneal macrophages primed by LPS and stimulated by SFAs [45]. In RAW264.7 macrophages, 500 μM PA increased apoptosis and reactive oxygen species production, which were reduced by the addition of OA [54]. PA, but not OA, increased TNF-α, IL-6, IL-1β, and MCP-1 in RAW264.7 cells [55], while OA increased the expression of Arg-1, CD206, and KLRT4 [56]. In vivo, OA reduced proinflammatory effects of SA by developing regulatory myeloid suppressive cells [57], characterized by accumulation of lipid droplets and production of nitric oxide (NO). Furthermore, OA reduced high-fat-diet–induced oxidative stress [58] and IL-1α production coupled with vascular inflammation and atherosclerosis [59]. These anti-inflammatory functions could be exerted by activation of microRNA let-7b, at least in the THP-1 monocytic cell line [60]. In a model of *Alternaria alternata*-induced pulmonary inflammation, the release of OA and LA from macrophages increased the activation of innate lymphoid cells type 2 (ILC2s), innate cells central to the development of type 2 inflammation, likely by binding to FFAR1 [61]. These data suggest opposing functions of OA in type 1 and type 2 inflammation. 

The role of other MUFAs (heptadecenoic acid, gadoleic acid, docosenoic acid, and tetracosenoic acid) in macrophage-mediated inflammation remains poorly understood.

### 2.4. Polyunsaturated Fatty Acids

**Figure 2:**
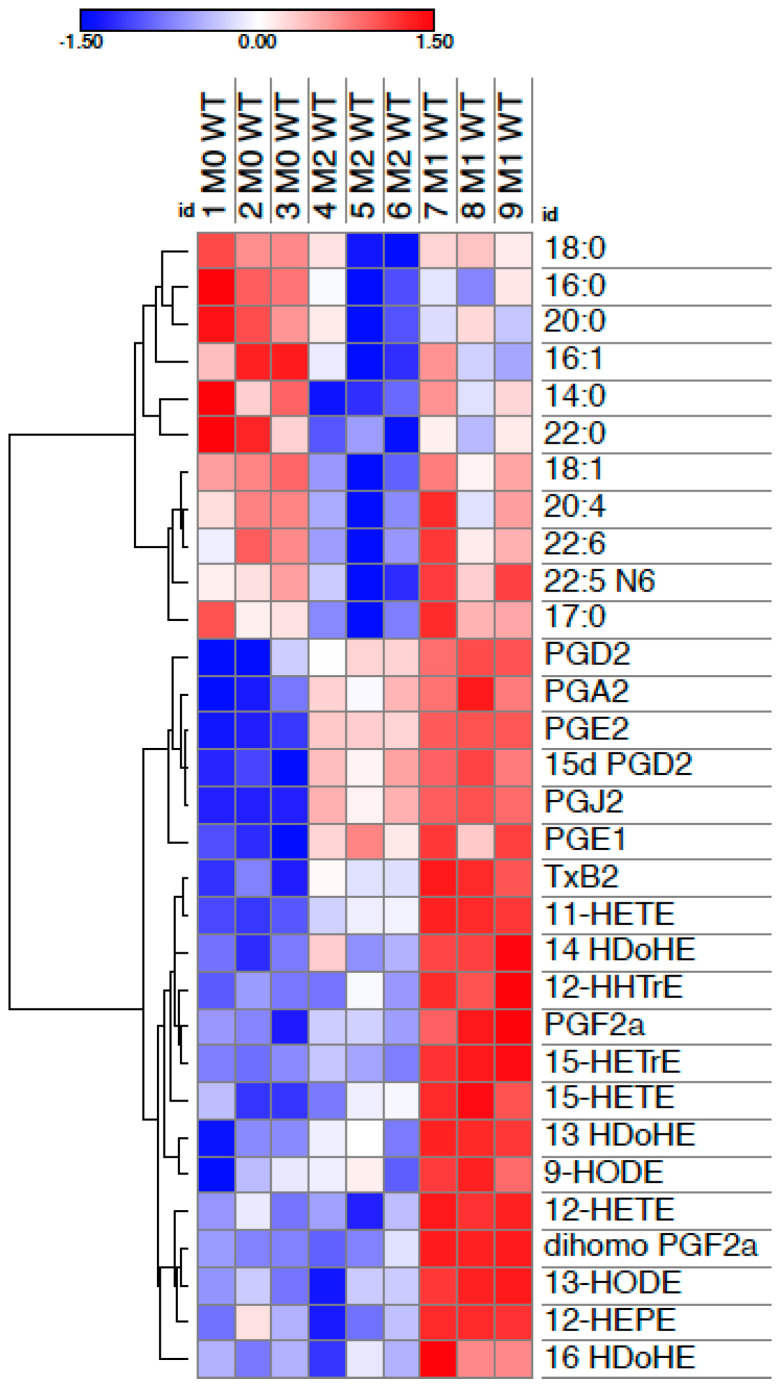
Hierarchical clustering of FAs and oxylipins generated in BM-macrophages activated with IL-4 (M2), LPS + IFNγ (M1), or unstimulated controls (M0). BM-macrophages were cultured as previously described [61,62]. FAs and oxylipins were analyzed in cell pellets and supernatants, respectively, and previously shown as pmol/mg of protein (FAs) or pmol/mL (oxylipins) [61,62]. Data are from 3 independent experiments; shown are mean intensities of lipids that reached *p* < 0.05 by Anova.

PUFAs liberated from membrane phospholipids by PLA_2_ enzymes can be metabolized into oxylipins through three major enzymatic pathways (Figure 1). To understand the pathways involved in the activation macrophages, here, we compare FAs and oxylipins liberated by wild-type mouse BM-macrophages unstimulated (M0) or stimulated with LPS + IFNγ (M1) or IL-4 (M2) [61,62]. The hierarchical clustering analysis shows that, compared to M1 or M2 macrophages, unstimulated BM-macrophages (M0) prefer to generate SFAs (Figure 2). Instead, M1 macrophages produce mainly COX- and 15-LOX oxylipins derived from omega-6 FAs arachidonic acid (AA) and LA and omega-3 DHA, suggesting a broad and potent activation. M2 macrophages mainly generated AA-derived COX products. However, given the heterogeneity of macrophages, it is likely that activation of macrophages, particularly in vivo, involves additional pathways and FAs.

#### 2.4.1. Linoleic Acid

LA is an essential FA from which are generated oxylipins and PUFAs. In a model of zymosan-induced peritoneal inflammation, dihydroxy-eicosatrienoic acids (DHETs) and dihydroxy-octadecenoic acid (DiHOME) derived, respectively, from AA and LA by the action of CYP450 epoxygenases limited recruitment of proinflammatory monocytes CX3CR1^low^, CCR2^high^, and CCL2^high^ [63], supporting the notion of a protective role of CYP450 derived epoxy-eicosatrienoic acids (EETs). However, 9-hydroxy-octadecadienoic acid (HODE) and 13-HODE, the LOX products of LA (Figure 1), increased the expression of the chemokine receptor CX3CR1 and reduced CCR2, favoring the adhesion of macrophages to coronary artery smooth muscle cells [64,65]. Additionally, 13-HODE, but not LA, stimulation of RAW 264.7 macrophages induced the expression of ATP-binding cassette transporter A1 (ABCA1) and increased cholesterol efflux in the presence of apolipoprotein A I [66]. Consistently OA and LA reduced cholesterol efflux in the presence of apolipoprotein A I by increasing ABCA1 degradation [67]. These studies may explain the protective function of LA-derived CYP450 products and the proinflammatory function of LA in atherosclerosis.

In type 2 inflammation, IL-4 upregulated 15-LOX in human monocyte-derived macrophages and 12/15-LOX in thioglycollate-elicited peritoneal macrophages. Indeed 15-HETE and 12/15-HETE were major products of macrophages activated by IL-4 in the presence of AA, while 13-HODE was the primary oxidative product of LA [68]. In a model of allergen-induced pulmonary inflammation, LA produced by macrophages induced activation of ILC2 expressing FFAR1 [61]. Another study supporting a proinflammatory role for LA in type 2 inflammation showed that IL-13 induced the release of 13-HODE from RAW 264.7 macrophages, thus suggesting that, at least in an ovalbumin (OVA) model of airway inflammation, macrophages contribute to epithelial injury [69]. Furthermore, LA was found to significantly increase in the serum obtained from patients with asthma COPD overlap [70].

#### 2.4.2. Gamma-Linolenic Acid and Dihomo-Gamma-Linolenic Acid

An elongation product of LA is Gamma-linolenic acid (GLA, C18-3 N6). Despite being an omega-6 FA, GLA reduced the inflammatory response in LPS-activated RAW 256.7 macrophages by reducing phosphorylation of extracellular signal-regulated kinase (ERK) 1/2 and c-Jun N-terminal kinase (JNK)-1 [71]. Another elongation product of LA is eicosadienoic acid (EDA, C20:2), which is rapidly metabolized to di-homo-gamma-linolenic acid (DGLA, C20:3n6) and AA (C20:4). Indeed, stimulation of RAW 264.7 macrophages with EDA resulted in increased AA incorporation into membrane phospholipids and increased PGE_2_ production [72]. On the contrary, the addition of DGLA to human monocyte-derived macrophages resulted in reduced chemokine production and macrophage migration [73]. Macrophages activated with LPS and DGLA produce PGE_1_ and PGD_1_ [74], molecules that could compete with the series 2 of lipids derived from AA. Therefore, the beneficial effects of DGLA in type 1 inflammation could be direct or indirect.

#### 2.4.3. Eicosatrienoic Acid

Eicosatrienoic acid (ETA, C20:3 N9) is an omega-9 fatty acid that is usually scarce unless there is a drastic reduction in the availability of AA. Indeed, following the reduction in AA, through the Lands cycle, the membrane was replenished with ETA N9 [31]. Although the function of ETA N9 in macrophage-driven inflammation needs to be further investigated, its presence indicates a deficiency in essential fatty acids [75].

#### 2.4.4. Arachidonic Acid

AA is the primary FA of the cell plasma membrane. The importance of AA in type 1 inflammation driven by macrophages has been recently validated using mice lacking the long-chain acyl-CoA synthetase 4 (ACSL4), which preferentially incorporated AA into membrane phospholipids [76]. In a model of zymosan-induced peritoneal inflammation, the absence of ACSL4, specifically in macrophages, reduced neutrophil recruitment and LTB_4_ and PGE_2_ generation. Recently, the lipid profile of human monocyte-derived macrophages obtained by negative selection, cultivated in M-CSF for 7 days and polarized with LPS + IFNγ, or IL-4, revealed a significant increase in TxB2 in LPS + IFNγ activation compared to controls and IL-4 [77]. These results align with data obtained in mouse BM-macrophages (Figure 2) since TxB2 was increased in M1 compared to M2 and M0. However, as described above about LA, EETs derived from AA by CYP450 epoxygenases (Figure 1) reduced the expression of M1 molecules (iNOS, MCP-1, IL-6) and increased the expression of M2 molecules (Arg-1, CD206) in human monocytic cell lines [78]. Consistently, we found that 20-carboxyAA (20CooHAA), an eicosanoid produced from AA by cytochrome P450 (CYP) omega-oxidases, was increased in IL-4 activated mouse BM-macrophages and human monocyte-derived macrophages in a Pla2g5-dependent fashion [62], confirming the importance of this pathway in IL-4 activation.

#### 2.4.5. Adrenic Acid

Although Adrenic acid (AdA, C22:4) is a FA derived by elongation of AA, AdA likely has specific functions in macrophages. Mouse peritoneal macrophages released AdA by the calcium-independent group VIA PLA_2_ following zymosan stimulation [79]. However, in inflammatory conditions, it is plausible that the liberation of AA from phospholipids is followed by its conversion into AdA [80] and subsequent re-acylation. Therefore, it has been hypothesized that AdA may function in the resolution phase of inflammation [81].

#### 2.4.6. Docosapentaenoic Acid

Docosapentaenoic acid (DPA, C22:5 n6) is formed from AA. Interestingly, incubation of RAW 264.7 cells with the 15-LOX derived oxidation product 17-HDPA reduced M1 markers iNOs and TNF-α mRNA, and increased scavenger receptor mRNA, an M2 marker [82].

### 2.5. Omega-3 PUFAs

ALA (C18:3 N3) and other omega-3 PUFAs have been reported to increase certain features of M2 macrophage activation. BM-macrophages activated with IL-4 and ALA showed an increase in Arg-1 mRNA and CD206 expression, likely through the activation of GPR40 (FFAR1) and PPARγ [83]. In the THP-1 human monocytic cell line activated with LPS and IFNγ, ALA reduced the production of inflammatory cytokines and increased the release of oxylipins derived from ALA and LA [84].

Docosahexaenoic acid (DHA, C22:6 N3) in vivo has antidiabetic functions and downregulates type 1 inflammation likely by acting through GPR120 [85] and, at least in part, through cytosolic PLA_2_ activation and PGE_2_ generation [86]. BM-macrophages isolated from mice lacking ELOVL2, the enzyme catalyzing the conversion of PUFA C22 to C24 and activated with LPS + IFNγ or IL-4, resulted in increased expression of M1 molecules (iNOS, IL-6, IL-12, CD86) and reduced expression of M2 molecules (CD206, CCL22, CCL17), but not Arg-1, respectively. The addition of DHA to macrophage cultures could restore these defects [87]. Furthermore, the anti-inflammatory effect of DHA on RAW 264.7 cells included reduced expression of the histone deacetylases, particularly HDAC3, 4, and 9 [88].

Interestingly, in BM-macrophages, DHA and eicosapentaenoic acid (EPA, 20:5N3) inhibited inflammasome activation and LPS-induced IL-1β secretion, likely signaling through both FFAR1 and FFAR4 [89]. EPA reduced cholesterol efflux in human THP-1 macrophages [90], which may help explain the protective effects of EPA in chronic inflammation, namely atherosclerosis [91]. Indeed, administration of a combination of DHA and EPA reduced atherosclerosis lesions in mice fed a western diet, and incubation of RAW 264.7 macrophages with EPA and DHA reduced MCP-1 (at low dose) and TNF-α (at high dose) [92]. Similarly, in another study, incubation of RAW 264.7 macrophages with EPA reduced NO and PGE_2_ production induced by LPS. These effects were partially reproduced (only reduction of NO) also with Eicosatrienoic acid N3 (ETA, C20:3 N3) [93]. Reduced NO production and nuclear factor κB (NFκB) activation were also obtained by the incubation of LPS-stimulated RAW 264.7 macrophages with Stearidonic acid (SDA 18:4 N3) [94]. It is likely that the incorporation in the macrophage plasma membrane of omega-3 FAs, including EPA, DHA, and Docosapentaenoic acid (DPA, C22:5 N3), shifts the balance of TLR-4 induced macrophage activation from AA-derived eicosanoids to omega-3-derived oxylipins [95].

## 3. Concluding Remarks

Since the first comprehensive lipid analysis of mouse macrophages [18], the availability of lipid mass spectrometry has drastically expanded the studies on lipid functions related to the many faces of macrophage activation in health and disease. Furthermore, the identification of bioactive lipids produced by macrophages in various conditions has improved our understanding of the role of lipids in the pathogenesis of many diseases.

Studies of macrophage development highlight the relevance of FAs in the biology of macrophages. Intriguingly, the differentiation of human monocytes into macrophages was associated with increased FA synthesis and desaturation, with PA being the most abundant FA in differentiated macrophages [96]. Furthermore, the same authors reported an increase in glycerophospholipids (PLs) during macrophage differentiation, particularly phosphatidylcholine (PC), as expected since the volume of macrophages is larger than monocytes. Inhibition of FA synthesis reduced not only PLs content but also the expression of CD11b, CD36, and Mrc1, markers of macrophage differentiation, and phagocytosis. These effects are likely due to both MUFAs and PUFAs, since OA, LA, DHA, and EPA increased CD36 expression in THP-1 monocyte-macrophages [97].

Type 1 inflammation requires that macrophages kill pathogens and produce cytokine and chemokines necessary to mount an inflammatory response. SFAs and oxylipins strongly contribute to type 1 inflammation, as supported by many studies (Table 1) and recent lipidomic analysis (Figure 2). IL-4 activation of macrophages is one of the many M2 “alternatives” to M1 macrophages. However, M2 macrophages seem to produce fewer FAs and oxylipins than M1 macrophages, although studies are still scarce (Table 1). Therefore, M2 macrophages could offer an alternative to type one inflammation by replacing M1 macrophages.

The combination of lipidomic, metabolomic, and transcriptomic will likely offer in the future a more comprehensive evaluation of the pathways engaged by FAs and oxylipins in type 1 and type 2 inflammation, thus allowing the identification of new therapeutic targets.

## Figures and Tables

**Table 1:** Fatty acids produced by activated macrophages and their role in inflammation.

Symbol	Name	Abbreviation	Effect on Inflammation	
			Type 1	Type 2
Medium-Chain Saturated Fatty Acids				
12:0	Lauric acid	LU	↑↓	?
14:0	Myristic acid	MA	↑	?
Uneven Saturated Fatty Acids				
15:0	Pentadecanoic acid	PdA	↓	?
17:0	Heptadecanoic or Margaric acid	MaA	?	?
Long-Chain Saturated Fatty Acids				
16:0	Palmitic acid	PA	↑	?
18:0	Stearic acid	SA	↑	?
20:0	Arachidic acid	ArA	↑	?
22:0	Docosanoic acid		?	?
23:0	Tricosanoic acid		?	?
24:0	Tetracosanoic acid		?	?
26:0	Hexacosanoic acid		?	?
Monounsaturated Fatty Acids				
16:1	Palmitoleic acid	PoA	↓	↑?
17:1	Heptadecenoic acid		?	?
18:1	Oleic acid	OA	↓	↑
20:1	Gadoleic acid		?	?
22:1	Docosenoic acid		?	?
24:1	Tetracosenoic acid		?	?
Polyunsaturated Fatty Acids Omega-6 and Omega-9				
18:2	Linoleic acid	LA	↑↓	↑
18:3 N6	Gamma-linolenic acid	GLA	↓	?
20:2	Eicosadienoic acid	EDA	↑	?
20:3 N6	Dihomo-gamma-linolenic acid	DGLA	↓	?
20:3 N9	Eicosatrienoic acid or mead acid	ETA N9	?	?
20:4	Arachidonic acid	AA	↑	↑
22:4	Adrenic acid	AdA	↑	?
22:5 N6	Docosapentaenoic acid	DPA N6	↓	↑
Polyunsaturated Fatty Acids Omega-3				
18:3 N3	Alpha-linolenic acid	ALA	↓	↑
18:4 N3	Stearidonic acid	SDA	↓	↑?
20:3 N3	Eicosatrienoic acid	ETA N3	↓	?
20:5 N3	Eicosapentaenoic acid	EPA	↓	?
22:5 N3	Docosapentaenoic acid	DPA N3	↓	?
22:6 N3	Docosahexaenoic acid	DHA	↓	?

↓ reduction in inflammation; ↑ increase in inflammation; ? not known.

**Table 2:** Functions of indicated FAs in type 1 inflammation.

Fatty Acid	Findings	Selected References
MA	Myristoylation of viral protein-4 (VP4) increased TLR2 aggregation with MyD88 in mouse BM-macrophages and chemokine production in human alveolar macrophages	[22]
	In human embryonic kidney (HEK)293 cells, myristoylation of TRIF-related adaptor molecule (TRAM), followed by its translocation to the plasma membrane, was essential for TLR4 signaling and LPS activation	[23]
LU	LU increased TLR signaling and COX-2 expression in RAW 264.7 macrophages	[24,25]
	LU increased killing of *Brucella abortus* in vitro and in vivo likely through GPR84	[26]
	Improved insulin resistance and reduced inflammation in THP-1 macrophages and in vivo	[27,28]
PdA	In a model of nonalcoholic steatohepatitis induced by methionine- and choline-deficient diet, administration of PdA reduced ceroid-laden macrophages	[30]
	PdA reduced reactive oxygen species in human hepatic cell line and production of type 1 proinflammatory cytokines and chemokines in peripheral blood mononuclear cells	[29]

## Abbreviations

Alpha-linolenic acid (ALA), arachidonic acid (AA), arginase-1 (Arg-1), cyclooxygenase-2 (COX-2), cytochrome P450 (CYP450), dihydroxy-eicosatrienoic acids (DHETs), dihydroxy-octadecenoic acid (DiHOME), linoleic acid (LA), 20-carboxyAA (20CooHAA), cysteinyl leukotrienes (CysLTs), fatty acids (FAs), desaturase (FAD), elongase (ELOVL), free fatty acid receptor (FFAR), group V phospholipase A_2_ (Pla2g5), hydroxy-octadecadienoic acid (HODE), innate lymphoid cells type 2 (ILC2s), lipoxygenase (LOX), Mannose receptor C-type 1 (Mrc1), monounsaturated fatty acids (MUFAs), nucleotide-binding oligomerization domain, leucine-rich repeat and pyrin domain-containing 3 (NLRP3), polyunsaturated (PUFAs), saturated fatty acids (SFAs), Transforming growth factor beta-1 (Tgf-β1).
